# Capacitive Sensor to Monitor Enzyme Activity by Following Degradation of Macromolecules in Real Time

**DOI:** 10.1007/s12010-019-03006-0

**Published:** 2019-04-24

**Authors:** Gizem Ertürk Bergdahl, Martin Hedström, Bo Mattiasson

**Affiliations:** 1 CapSenze Biosystems AB, Lund, Sweden; 2 Department of Biotechnology, Lund University, Lund, Sweden

**Keywords:** Capacitive sensor, Degradation, Enzymatic activity, Protease

## Abstract

A capacitive sensor was developed to analyze the presence and enzymatic activity of a model protease from standard solutions by following the degradation of the substrate in real time. The enzyme was chosen based on its specific digestion of the hinge region of immunoglobulin G (IgG). Real-time enzyme activity was monitored by measuring the change in capacitance (∆C) based on the release of IgG fragments after enzymatic digestion by the enzyme. The results indicated that the developed capacitive system might be used successfully for label-free and real-time monitoring of enzymatic activity of different enzymes in a sensitive, rapid, and inexpensive manner in biotechnological, environmental, and clinical applications.

**Electronic supplementary material:**

The online version of this article (10.1007/s12010-019-03006-0) contains supplementary material, which is available to authorized users.

## Introduction

Enzyme activity assays are generally based on measuring either the consumption of the substrate or the formation of the product over time [1]. There are various methods developed to measure these concentrations and thereby to monitor the enzymatic activity [2]. One of them is end-point enzyme assays like chromatographic and radiometric assays. On the other hand, there are also kinetic enzyme assays including spectrophotometric, fluorometric, calorimetric, chemiluminescent, and light scattering assays which are more convenient and easier than the end-point enzyme assays [3, 4]. However, these methods are generally based on the use of substrates labeled with chromatophores, fluorophores, or radioactive markers [5]. These assays may be sensitive but they require multi-step analysis with labeling reagents that cause interference with the assay [6]. Real-time analysis of the activity in terms of binding kinetics is still limited [7].

The model protease (FabRICATOR®) that we used in our study represents a group of enzymes where activity assays are cumbersome. For this enzyme, a typical assay involves incubation of the enzyme with the substrate, IgG, and then analysis of the formed products using either electrophoresis or chromatographic separation [8–10]. There are several enzymes especially proteases with similar assay problems and many of these enzymes are produced by pathogenic microorganisms. The proteases are important since they might be used as indicators for the presence of a certain microorganism, but then one needs fast and efficient procedures for assaying the enzymatic activity.

Recently, there has been an increasing interest in the development of label-free detection methods which include real-time sensing in a sensitive, easy, and fast manner [11]. Many label-free detection strategies have been reported including optical and electrochemical sensing for various biological compounds including antibodies, antigens, toxins, biomarkers, proteins, and nucleic acids [12–16]. Capacitive biosensors are the type of label-free electrochemical biosensors which are usually referred to as a subcategory of impedance biosensors in which the change in capacitance value (∆C) is measured directly [17]. In the capacitive sensing, the affinity interaction between the biorecognition surface and the target analyte results in a change in the capacitance at the solid-liquid interface due to the displacement of the counter ions around the capacitive electrode [18]. If higher amounts of target molecules are bound to the affinity layer, the achieved displacement and the decrease in the registered capacitance are getting higher. The main advantages of capacitive systems are the ease of detection and higher sensitivity when compared to other label-free biosensors [18, 19]. Over the last years, capacitive biosensors have been attractive in clinical, environmental, and biotechnological applications including analysis of toxins, proteins, nucleic acids, and pharmaceutics [20–26].

In the study reported here, a capacitive biosensor was developed for detecting the enzyme activity of a protease by following the degradation of the substrate in real time. To our knowledge, measuring the enzyme activity by following the degradation of substrates in real time via capacitive biosensors is reported for the first time in the literature by this study.

## Materials and Methods

### Materials

3-Aminophenylboronic acid monohydrate (APBA), *N*-hydroxysuccinimide sodium salt (NHS), 1-(3-dimethylaminopropyl)-3-ethylcarbodiimide hydrochloride (EDC), sodium carboxymethylcellulose (Na-CMC), and tyramine (99%, HOC_6_H_4_CH_2_CH_2_NH_2_) were obtained from Sigma-Aldrich (Steinheim, Germany). Human gamma globulin (IgG) was purchased from Octapharma AB (Stockholm, Sweden). Recombinant protein A (rProA) was supplied from Indienz AB (Billeberga, Sweden). 1-Dodecanethiol was obtained from Aldrich (Deisenhofen, Germany). Glutaraldehyde (50%, *V*/*V*) was purchased from Fluka (Buchs, Switzerland). FabRICATOR (IdeS)® was supplied from Genovis AB (Lund, Sweden). All other chemicals used were of analytical grade. All buffers were prepared with water treated with a reverse osmosis step with a Milli-Q system from Millipore (Bedford, MA, USA). Prior to use, all buffers were filtered through Millipore filter (pore size 0.22 μm) and degassed for 1 h.

### Preparation of Capacitive Electrodes

In order to develop an assay for measuring real-time enzyme activity by monitoring the degradation of substrates, two different immobilization strategies were used for the preparation of capacitive electrodes.

The first strategy was based on the use of substrate (IgG) covalently immobilized to the electrode surface and then to monitor the degradation. The second strategy involved a reversible immobilization method such that after the assay, the electrode could be regenerated and reused.

**Scheme 1:**
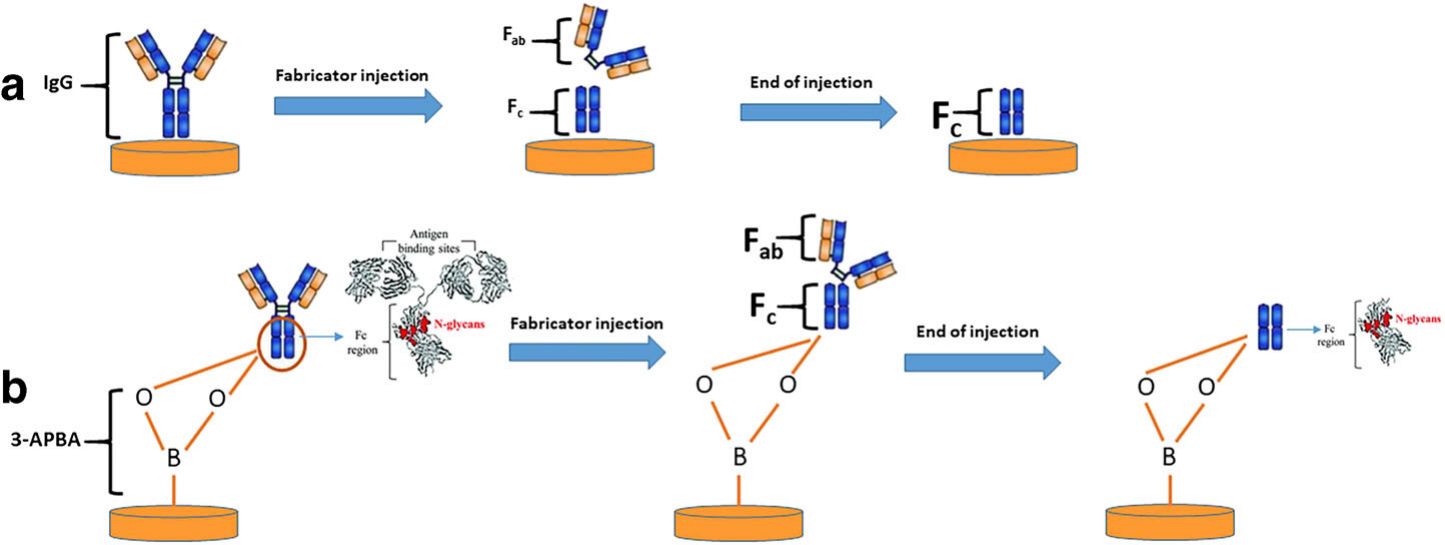
Preparation of the capacitive electrodes by using different strategies and activity of the enzyme on these electrodes, **a** IgG-immobilized electrodes and **b** APBA-immobilized electrodes

Preparation of the capacitive electrodes by using these strategies and activity of the enzyme on these electrodes are schematically shown in Scheme 1.

#### Preparation of IgG-Immobilized Capacitive Electrodes

For the immobilization of IgG, in the first step, the electrodes were subsequently cleaned with ethanol, de-ionized water, acetone, de-ionized water, and acidic Piranha solution [3:1, H_2_SO_4_ (95%):H_2_O_2_ (30%), *v*/*v*] respectively for 10 min in each step, in an ultrasonic cleaner. Then, plasma cleaning (Mod. PDC-3XG, Harrick, NY, USA) was applied to the electrodes for 20 min. Afterwards, electro-polymerization of tyramine was performed by using a cyclic voltammetry (CV) in ethanolic solution of 10 mM tyramine with a set potential range of 0–1.5 V (vs Ag/AgCl) and a scan rate of 50 mV s^−1^ for 15 scans as described before [27]. By this way, free primary amino groups were introduced on the surface of the electrode. Then, the electrodes were rinsed with distilled water and dried with nitrogen gas. In the next step, for the activation of amino groups on the surface, the electrodes were immersed in 5.0% (*v*/*v*) glutaraldehyde solution in 10 mM phosphate buffer (pH 7.4) for 1 h at room temperature. Then, electrodes were rinsed with distilled water and dried with nitrogen gas.

In the last step, electrodes were immersed overnight at 4 °C in IgG solution (0.1 mg mL^−1^) prepared in 10 mM phosphate buffer (pH 7.4). Finally, to cover pinholes in the insulating layer of the gold surface, they were kept in 10 mM of 1-dodecanethiol in ethanol for 20 min. 1-Dodecanethiol treatment does not affect the immobilized protein on the surface and only interacts with the pinholes on the surface. Therefore, it has been used for this purpose in so many previous reports where capacitive sensors are used for detection [28–31].

#### Preparation of 3-Aminophenylboronic Acid (APBA)-Immobilized Capacitive Electrodes

In the first step, gold electrodes were cleaned by using the same strategy described above in the “Preparation of IgG-Immobilized Capacitive Electrodes” section. In the next step, electro-polymerization of tyramine was performed by cyclic voltammetry (CV) in ethanolic solution of 10 mM tyramine with a set potential range of 0–1.5 V (vs. Ag/AgCl) and a scan rate of 50 mV s^−1^ for 15 scans. Then, sodium carboxymethyl cellulose was dissolved in 0.05 M morpholinoethanesulfonic acid (MES) buffer (pH 6.0) at 1.0% (*w*/*v*). Poly-tyramine-coated electrodes were immersed in this solution for 1 h at room temperature. By this way, carboxyl groups were introduced on the surface of the electrode. Same method has been used successfully for the 3-APBA modification of capacitive gold electrode surface for saccharide modification in our previous study [32].

In the next step, for the activation of carboxyl groups, electrodes were immersed in 1 mL of 0.05 M 1-(3-dimethylaminopropyl)-3-ethylcarbodiimide hydrochloride and 1.0 mL of 0.03 M N-hydroxysuccinimide sodium salt in MES buffer (pH 6.0) for 2 h. *N*-Hydroxysuccinimide-activated carboxylic groups were then allowed to bind with the primary amino groups of 3-aminophenylboronic acid (40 mM) in phosphate buffer (10 mM, pH 7.0) overnight, at room temperature [32]. Finally, to cover bare parts of the gold surface, they were kept in 10 mM of 1-dodecanethiol in ethanol for 20 min.

### Surface Characterization of Electrodes with Cyclic Voltammetry

APBA-immobilized electrodes were used for surface characterization of capacitive electrodes with CV. CV experiments were carried out using a potentiostat/galvanostat (Autolab PGSTAT12, EcoChemie, Utrecht, The Netherlands). All electrochemical measurements were conducted in a standard three-electrode flow cell. A platinum wire and a commercial Ag/AgCl electrode were used as the counter and the reference electrodes, respectively. APBA-immobilized electrode served as the working electrode. A solution of KCl (0.1 M) containing 0.1 M potassium ferricyanide was used as the electrolyte solution. Cyclic voltammetry was performed by sweeping the potential between −0.3 and 0.8 V at a sweep rate of 0.1 V s^−1^.

### Monitoring Enzyme Activity with the Developed Capacitive Systems

Capacitive measurements were performed with an automated flow injection system (CapSenze Biosystems AB, Lund, Sweden). The immobilized electrodes were inserted into the electrochemical flow cell, and the capacitive measurements were performed via current pulse method as described previously [27].

#### Capacitive Measurements with IgG-Immobilized Electrodes

Enzyme activity was evaluated using the IgG-modified capacitive electrode by measuring the change in capacitance (∆C) based on the release of fragments of IgG formed during enzymatic digestion by FabRICATOR®. The analysis started with the injection of a regeneration solution (25 mM glycine-HCl, pH 2.5) into the system for the re-equilibration of the surface. After injection of the running buffer (10 mM phosphate, pH 7.4) to get a stable baseline, enzyme solution was injected into the capacitive system. The capacitance values were determined before and after enzymatic digestion of immobilized IgG molecules. The difference in capacitance was defined as ∆C. The enzymatic digestion of immobilized IgG on the electrode surface resulted in an increase of the registered capacitance. The fragments of IgG formed after enzymatic digestion were F_(ab)2_ and F_c_. These fragments were removed from the surface after enzymatic digestion which resulted in a decrease in the achieved displacement of the counter ions around the electrode. According to the assaying principle of capacitive biosensors, this results in an increase in the registered capacitance signal. This change (∆C) was calculated automatically by the system. The flow rate was 100 μL min^−1^, and the volume of the injected enzyme was 250 μL in the analysis.

The enzyme cleaved the hinge region of IgG to produce an F_(ab)2_ fragment and two fragments of F_c_. Therefore, the electrode was not reusable. To prevent the electrode being disposable and to be able to obtain a relatively inert surface, it was decided to monitor enzyme activity by using electrodes with reversible immobilized IgG which could be regenerated, recharged, and reused.

#### Capacitive Measurements with APBA-Immobilized Electrode

As a first step, a regeneration solution (25 mM glycine-HCl, pH 2.5) was injected into the system. In the second step, samples containing IgG were injected into the system to provide binding of IgG to APBA on the surface. After injection of the running buffer (10 mM phosphate, pH 7.4) to get a stable baseline, standard solutions of different concentrations of IgG (10^−13^ M–10^−9^ M) were injected sequentially. Binding of IgG to the electrode surface resulted in a decrease of the registered capacitance.

In the second step, enzyme activity was evaluated by measuring the change in capacitance (∆C) based on the release of fragments of IgG formed after enzymatic digestion on the IgG-bound APBA-immobilized electrode surface. ∆C value was monitored after IgG bound to the surface and after enzyme activity on the IgG-bound surface. Finally, regeneration of the system was utilized prior to the next analysis cycle.

## Results and Discussion

IgG-immobilized capacitive electrode represented the simplest way to be used for assaying the enzyme activity. Since the FabRICATOR® specifically digests the hinge region of IgG, producing a F_(ab)2_ fragment and two fragments of F_c_, it resulted in the digestion of the IgG molecules immobilized on the surface. These electrodes were thus disposable. Therefore, in order to make reusable capacitive electrodes, a different strategy was utilized by applying reversible immobilization of the IgG molecules via APBA.

For this purpose, APBA was immobilized on the capacitive sensor surface. In the next step, immunoglobulin G (IgG) was injected into the system and allowed to bind to APBA through N-glycans on the F_c_ region. Then, standard solutions of enzyme were injected into the system, and the enzyme activity was measured by following the ∆C before and after enzymatic digestion on the surface.

### Surface Characterization of Electrodes with CV

**Fig. 1:**
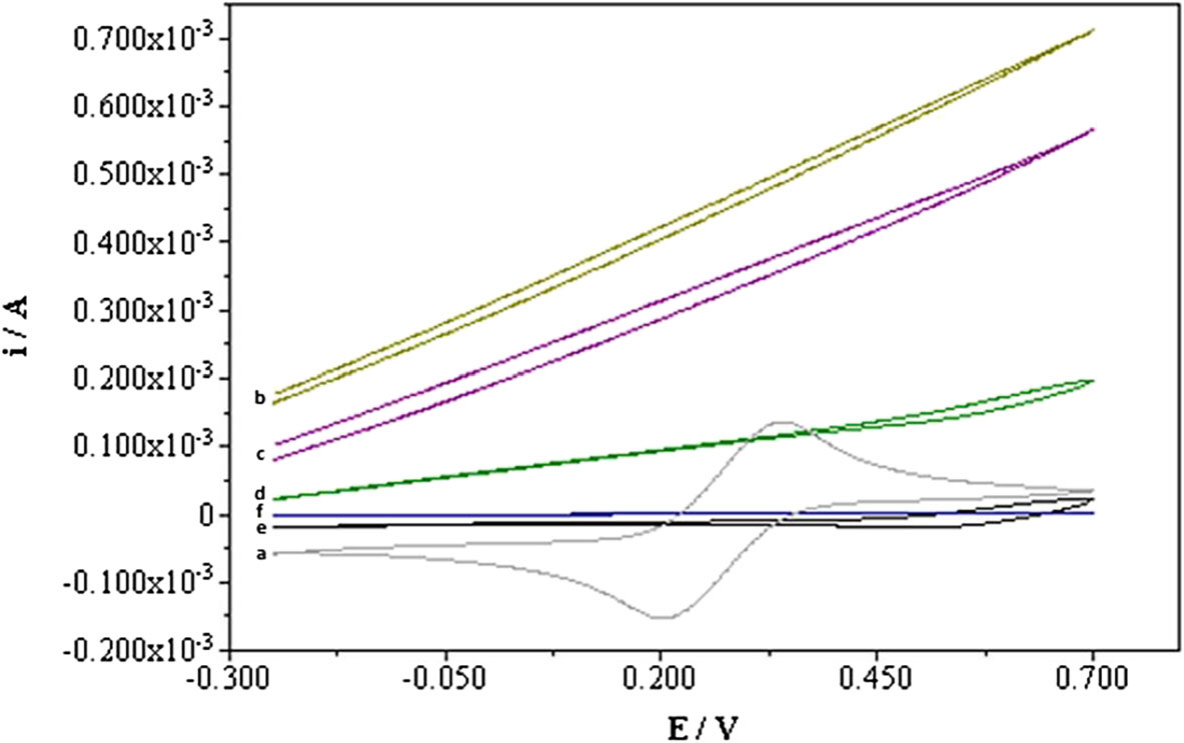
Characterization of electrodes with cyclic voltammogram recorded in a solution of 100 mM KCl containing 100 mM K_3_[Fe(CN)_6_] using bare gold electrode (a, gray), gold electrode after electro-polymerization of tyramine (b, yellow), gold electrode after carboxymethyl cellulose (CMC) activation (c, purple), gold electrode after carboxylic group of CMC was activated with EDC/NHS (d, green), gold electrode after NHS-activated groups were exposed and bonded to 3-APBA (e, black), and gold electrode after treatment with 1-dodecanethiol (f, blue). The scan range was from −0.3 to 0.8 V (vs. Ag/AgCl) at a scan rate of 0.1 V s^−1^

Proper insulation of the electrode surface is crucial in capacitive biosensors. The degree of insulation can be monitored by cyclic voltammetry of electro-active species in a contacting aqueous solution. Electrode with immobilized APBA was used to investigate the electrochemical characterization of the surface after each modification step (Fig. 1). The anchor layer should be completely insulated for a successful assay. The coating of the electrode surface with thin and non-conducting polymers such as tyramine by electrochemical deposition is an alternative to self-assembled monolayers (SAMs) with the advantages of the ease of their preparation and possibility of precise and reproducible mass production with the desired thickness [33]. These polymers also provide functional groups for coupling to the biomolecules. As seen from Fig. 1, the clean and bare gold surface showed large redox peaks (a, gray). The amplitude decreased after tyramine electro-polymerization (b, yellow) and carboxymethyl cellulose activation (c, purple) on the gold surface. Further reduction of the redox peaks was observed when the carboxylic groups of CMC were activated with EDC/NHS (d, green). As seen from the figure, the peaks almost disappeared when the activated groups were exposed and reacted with 3-APBA (e, black). Finally, treatment with 1-dodecanethiol led to disappearance of the redox peaks and lowered the current responses in both anodic and cathodic processes which resulted in a highly blocked interface (f, blue). The long aliphatic chain of 1-dodecanethiol is used to obtain a completely insulated electrode surface. The electrochemical results indicate that a complete and tight coverage of the APBA-modified electrode was provided and the electrode could be used for analysis.

### Monitoring Real-Time Enzyme Activity with Capacitive Electrodes

#### Capacitive Measurements with IgG-Immobilized Electrodes

**Fig. 2:**
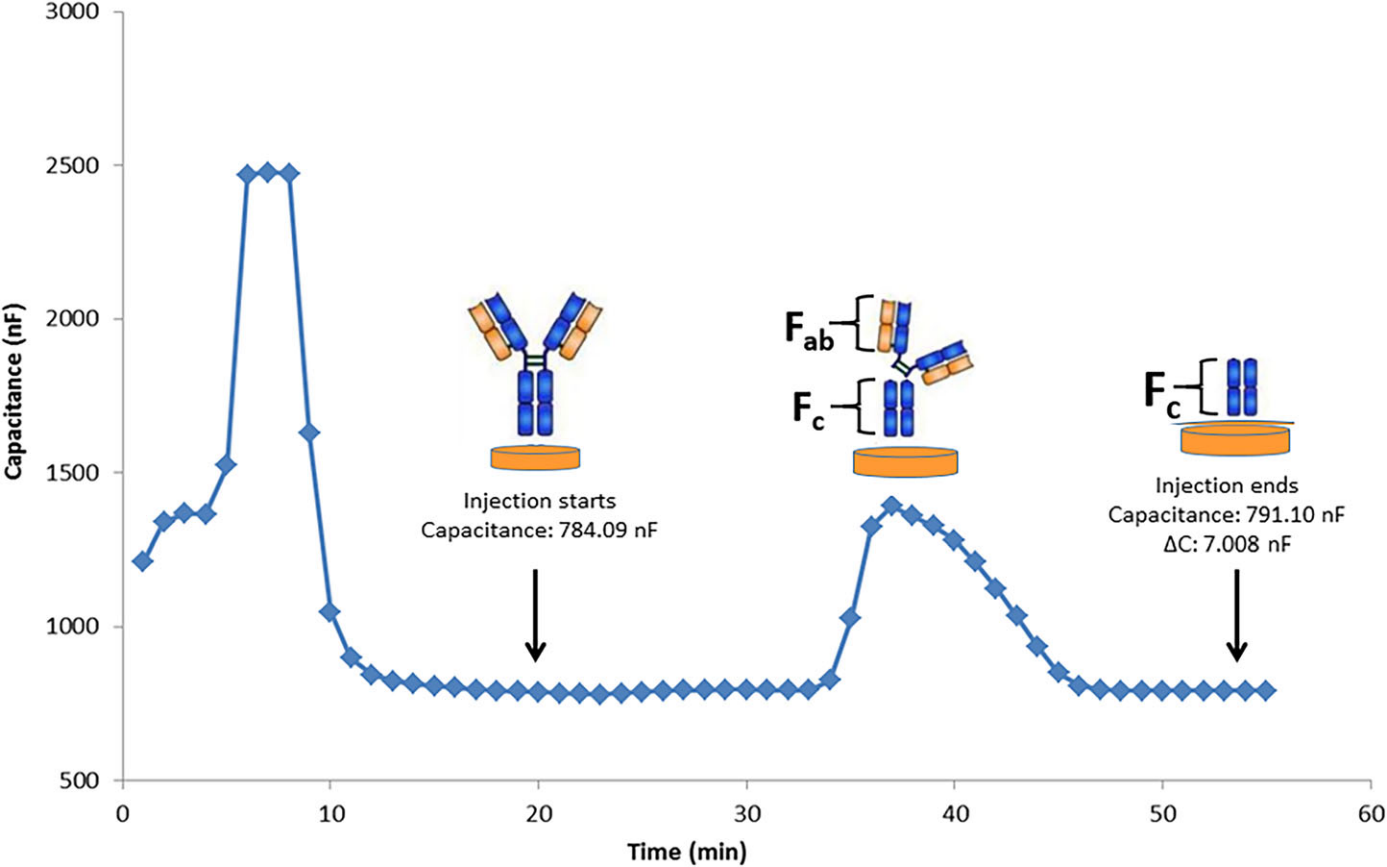
Actual sensorgram showing the capacitance change after enzyme injection (1.0 U) into the IgG-immobilized capacitive system (running buffer 10 mM phosphate, pH 7.4; regeneration buffer 25 mM glycine-HCl, pH 2.5). [Capacitance value (C) recorded before FabRICATOR® injection 784.09 ± 70.74 nF, capacitance value recorded after FabRICATOR® injection 791.10 nF ± 41.47, change in capacitance (∆C) 7.008 ± 3.59 nF]

IgG molecules, which were immobilized on the sensor surface, were cleaved into F_(ab)2_ and fragments of F_c_ upon FabRICATOR® treatment, and the fragments were removed from the surface as seen in Fig. 2. According to the assaying principle of capacitive biosensors, this resulted in an increase in the registered capacitance signal, in this case from 784.09 ± 70.74 nF to 791.10 ± 41.47 nF. Figure 2 shows the actual sensorgram obtained after enzyme injection into the IgG-immobilized capacitive system. This result shows that after enzyme injection into the capacitive system, ∆C value increased (∆C = 7.008 ± 3.59 nF), as expected. Therefore, by measuring the change in capacitance before and after enzyme injection made it possible to monitor the enzyme activity in real time. However, the digestion of immobilized IgG molecules on the electrode resulted in the electrode could not be re-used and made it necessary to perform each analysis with new electrodes. Therefore, in order to avoid the capacitive electrodes being disposable, a different strategy was utilized by using reversible immobilization of the IgG molecules. 3-Aminophenylboronic acid was used as the capturing agent/linker to prepare the sensor surface.

#### Capacitive Measurements with APBA-Immobilized Electrodes

**Fig. 3:**
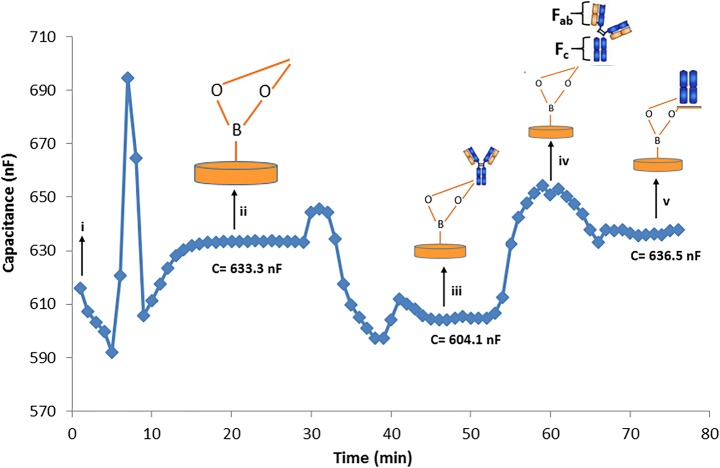
Actual sensorgram that shows the capacitance after IgG (10^−5^ M) and enzyme (1.0 U) injection, respectively, into the APBA-immobilized capacitive system (running buffer 10 mM phosphate, pH 7.4; regeneration buffer 25 mM glycine-HCl, pH 2.5) [i: injection of regeneration buffer, ii: injection of IgG, iii: end of IgG injection and equilibration of the system with running buffer, iv: FabRICATOR® injection, v: end of FabRICATOR® injection and equilibration of the system with running buffer, C before IgG injection 633.3 ± 24.03 nF, C after IgG and before FabRICATOR® injection 604.1 ± 15.6 nF, and C after FabRICATOR® injection 636.5 ± 11.59 nF]

Before assaying the enzyme activity with APBA-immobilized electrodes, standard IgG solutions in different concentrations (10^−13^ M–10^−9^ M) were prepared in the running buffer and sequentially injected into the capacitive system to obtain APBA-IgG binding via the N-glycans on the F_c_ region of IgG on the surface. Triplicate measurements were performed for each analysis. After injection of the sample and equilibration period in totally 15 min, regeneration buffer was injected into the system for 2.5 min to regenerate the surface and re-equilibration of the system took place during 45 min before a second injection period started (Fig. 3-i). Binding of IgG (Fig. 3-ii) on the electrode surface with immobilized APBA caused a consequent decrease in capacitance as shown in Fig. 3 from 633.3 ± 24.03 nF (Fig. 3-ii) to 604.1 ± 15.6 nF (Fig. 3-iii).

In order to measure the enzymatic activity, enzyme solutions were injected directly into the IgG-bound APBA-immobilized capacitive system (Fig. 3-iv). ∆C value was monitored again after enzyme injection which resulted in the capacitance value increased from 604.1 ± 15.6 nF (Fig. 3-iii) to 636.5 ± 11.59 nF (Fig. 3-v) after the enzyme activity. Since the formed fragments of IgG due to the enzyme activity were removed from the surface, it resulted in an increase in the registered capacitance signal.

The results clearly demonstrated that reversible immobilization makes it possible to reuse the electrodes and thereby making the process easier, faster, and cheaper.

### Detection of Real-Time Enzyme Activity from the Fermentation Medium

Fermentation medium is a specially designed complex growth medium which supplies the nutrients required by the organisms. The medium contains a carbon source, a nitrogen source, water, salts, and micronutrients. In order to investigate the usability and reliability of the developed system, recovery experiments were performed by detection of enzyme spiked in fermentation medium. For this purpose, IgG (10^−5^ M) is first injected into the capacitive system. Then, standard enzyme solutions were prepared in the fermentation medium and injected into IgG-bound capacitive system.

The recovery results are shown in Table 1. A recovery was obtained in the range of approximately 87% and 95%. In the study, the obtained recovery values demonstrate the accuracy and applicability of the developed capacitive system to measure enzyme activity even in the complex samples such as fermentation medium.

**Table 1:** Recovery results for enzyme spiked in fermentation medium (*n* = 3)

Samples	Added enzyme (μg/mL)	Found enzyme (μg/mL)	Recovery (%)
Sample number 1	0.1	0.087 ± 0.003	87
Sample number 2	0.2	0.18 ± 0.02	90
Sample number 3	0.3	0.26 ± 0.03	87
Sample number 4	0.4	0.38 ± 0.04	95

## Conclusion

Capacitive sensor was shown as a promising tool to measure the activity of enzymes with hydrolytic function with a total assay time of 45 min. This method can be of special interest in reactions especially where there are no simple assays available.

The choice of immobilization method is important while designing the experiments. It was demonstrated that reversible immobilization based on affinity interactions of the substrate molecule with immobilized affinity binders represents attractive means to make the electrodes reusable. Minimized non-specific interactions with the sensor surface are essential when the sensor is used for analysis in complex media. In the last part of the study, measuring the enzymatic activity from spiked fermentation media clearly showed the usability of the developed assay also in complex environments with a recovery in the range of approximately 87% and 95%.

## Electronic Supplementary Material

ESM 1 (DOCX 194 kb)
