# Supplementary material for: Capacitive Sensor to Monitor Enzyme Activity by Following Degradation of Macromolecules in Real Time
Source: Appl Biochem Biotechnol. 2019 Apr 24;189(2):374–83. doi: 10.1007/s12010-019-03006-0 (PMC6754820; doi:10.1007/s12010-019-03006-0)
Supplement: Supplementary file 1 — (DOCX 194 kb) [file 12010_2019_3006_MOESM1_ESM.docx]

**SUPPLEMENTARY INFORMATION**

**Capacitive sensor to monitor enzyme activity by following degradation of macromolecules in real-time**

**Gizem Ertürk Bergdahl^1.2*^, Martin Hedström^1.2^ and Bo Mattiasson^1.2^**

^1^ CapSenze Biosystems AB, Lund, Sweden

^2^Department of Biotechnology, Lund University, Sweden


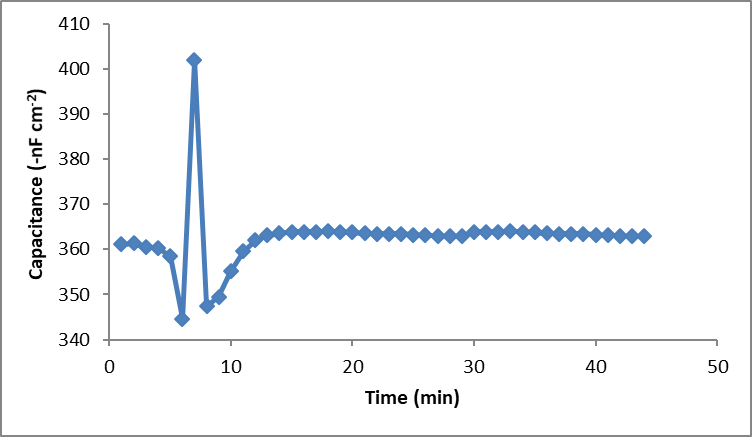


Start of injection

**Figure SI-1.** Injection of FabRICATOR enzyme directly onto the tyramine modified surface (before IgG immobilization)


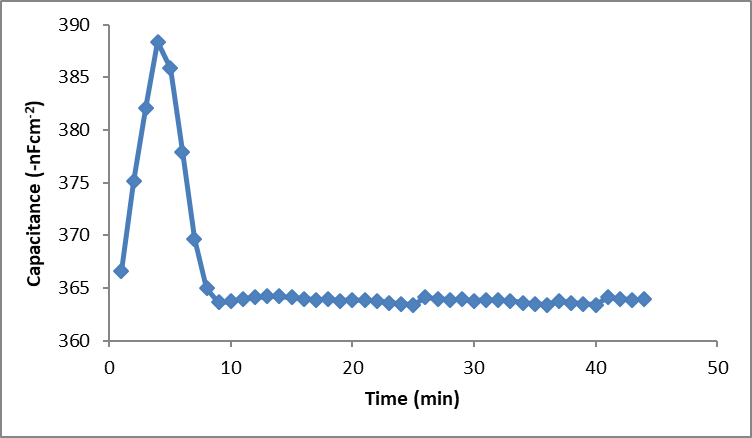


Start of injection

**Figure SI-2.** Injection of FabRICATOR enzyme directly onto the 3-APBA modified surface (before IgG injection)

As shown in Figure SI-1 and SI-2, the sensor did not give any significant response to the Fabricator enzyme when there was not any substrate (IgG) on the surface.

**Figure SI-3.** Actual sensorgram that shows the capacitance change after enzyme (0.1 µg/mL) injection. (running buffer: 10 mM phosphate, pH: 7.4; regeneration buffer: 25 mM glycine-HCl, pH: 2.5). [Capacitance value (C) recorded before FabRICATOR® injection: 363.3 nF, Capacitance value recorded after FabRICATOR® injection: 367.6 nF, Change in capacitance (∆C): 4.3 nF]

**Figure SI-4.** Actual sensorgram that shows the capacitance change after enzyme (0.2 µg/mL) injection. (running buffer: 10 mM phosphate, pH: 7.4; regeneration buffer: 25 mM glycine-HCl, pH: 2.5). [Capacitance value (C) recorded before FabRICATOR® injection: 363.3 nF, Capacitance value recorded after FabRICATOR® injection: 369.6 nF, Change in capacitance (∆C): 6.3 nF]

**Figure SI-5.** Actual sensorgram that shows the capacitance change after enzyme (0.3 µg/mL) injection. (running buffer: 10 mM phosphate, pH: 7.4; regeneration buffer: 25 mM glycine-HCl, pH: 2.5). [Capacitance value (C) recorded before FabRICATOR® injection: 365.3 nF, Capacitance value recorded after FabRICATOR® injection: 374.3 nF, Change in capacitance (∆C): 9.0 nF]

**Figure SI-6.** Actual sensorgram that shows the capacitance change after enzyme (0.4 µg/mL) injection. (running buffer: 10 mM phosphate, pH: 7.4; regeneration buffer: 25 mM glycine-HCl, pH: 2.5). [Capacitance value (C) recorded before FabRICATOR® injection: 363.3 nF, Capacitance value recorded after FabRICATOR® injection: 372.6 nF, Change in capacitance (∆C): 9.3 nF]
